# Acute neural effects of fluoxetine on emotional regulation in depressed adolescents

**DOI:** 10.1017/S0033291722001805

**Published:** 2023-07

**Authors:** Liliana P. Capitão, Robert Chapman, Nicola Filippini, Lucy Wright, Susannah E. Murphy, Anthony James, Philip J. Cowen, Catherine J. Harmer

**Affiliations:** 1 Oxford University Department of Psychiatry and Oxford Health Biomedical Research Centre, Oxford, UK; 2 Oxford Health NHS Foundation Trust, Oxford, UK; 3 IRCCS San Camillo Hospital, Venice, Italy

**Keywords:** adolescents, antidepressants, emotional regulation, major depressive disorder, neuroimaging

## Abstract

**Background:**

Adolescent major depressive disorder (MDD) is associated with disrupted processing of emotional stimuli and difficulties in cognitive reappraisal. Little is known however about how current pharmacotherapies act to modulate the neural mechanisms underlying these key processes. The current study therefore investigated the neural effects of fluoxetine on emotional reactivity and cognitive reappraisal in adolescent depression.

**Methods:**

Thirty-one adolescents with MDD were randomised to acute fluoxetine (10 mg) or placebo. Seventeen healthy adolescents were also recruited but did not receive any treatment for ethical reasons. During functional magnetic resonance imaging (fMRI), participants viewed aversive images and were asked to either experience naturally the emotional state elicited (‘Maintain’) or to reinterpret the content of the pictures to reduce negative affect (‘Reappraise’). Significant activations were identified using whole-brain analysis.

**Results:**

No significant group differences were seen when comparing Reappraise and Maintain conditions. However, when compared to healthy controls, depressed adolescents on placebo showed reduced visual activation to aversive pictures irrespective of the condition. The depressed adolescent group on fluoxetine showed the opposite pattern, i.e. increased visuo-cerebellar activity in response to aversive pictures, when compared to depressed adolescents on placebo.

**Conclusions:**

These data suggest that depression in adolescence may be associated with reduced visual processing of aversive imagery and that fluoxetine may act to reduce avoidance of such cues. This could reflect a key mechanism whereby depressed adolescents engage with negative cues previously avoided. Future research combining fMRI with eye-tracking is nonetheless needed to further clarify these effects.

## Introduction

Depression is a common mental health condition in adolescence (Kovacs, Goldston, & Gatsonis, 1993; Weissman et al., 1999). Clinically, young people with depression often report problems in regulating their emotions, in addition to the typical symptoms of low mood and irritability. Compared to non-depressed peers, research shows that adolescents with depression show increased reactivity to negative stimuli and are less able to engage in cognitive reappraisal, i.e. the ability to change the interpretation of a negative situation in order to reduce negative affect (Schäfer, Naumann, Holmes, Tuschen-Caffier, & Samson, 2017). These difficulties may have a particularly deleterious impact when experienced in adolescence, as this age period is characterised by numerous challenges and situations that can be perceived as highly negative or distressing, such as peer rejection and the need to cope with new emotions and feelings (Ahmed, Bittencourt-Hewitt, & Sebastian, 2015; Marston, Hare, & Allen, 2010; Sebastian, Viding, Williams, & Blakemore, 2010). For instance, in response to a friend not returning a text message, or cancelling a plan last minute, depressed adolescents are more likely to think that their friend does not like them, rather than consider alternative interpretations such as ‘they must be busy’ or ‘something must have happened that made them cancel their plans’. This reduced ability to reframe negative and ambiguous situations is known to intensify the experience of depression. The development of effective emotional regulation strategies such as cognitive reappraisal is therefore an important target for psychological and pharmacological treatments in adolescent depression.

Although cognitive reappraisal is an important construct, it is difficult to measure objectively because self-report measures are often confounded by negative biases in memory, difficulties in interoception and demand characteristics. An alternative method of indexing the neural circuits responsible for emotional regulation through reappraisal uses task-based functional magnetic resonance imaging (fMRI). In depressed adults, these paradigms have involved the presentation of negative/aversive pictures with and without the use of trained reappraisal strategies (Phan et al., 2005). Neural activity during cognitive reappraisal is then compared with a maintain/attend condition where participants are required to naturally experience the negative pictures depicted. Adults with depression typically show heightened activity in limbic-subcortical regions that are linked to the generation of emotional responses in the maintain/attend blocks, combined with reduced activity in regions that mediate ‘top down’ cognitive control, particularly the dorsolateral prefrontal cortex (DLPFC), during cognitive reappraisal (Disner, Beevers, Haigh, & Beck, 2011; Erk et al., 2010; Joormann & Stanton, 2016; Price & Drevets, 2012). There is some evidence suggesting that, similar to adults, depressed adolescents show heightened amygdala reactivity to negative pictures, combined with insufficient control from the still immature prefrontal cortex (PFC) (LeWinn et al., 2018; Platt et al., 2015). However, this finding is not always replicated, with some studies suggesting that adolescents with depression show preserved or even enhanced PFC activity during cognitive reappraisal (McRae, Rekshan, Williams, Cooper, & Gross, 2014; Young, Sandman, & Craske, 2019). The neural circuits supporting cognitive reappraisal undergo significant maturation during adolescence (Powers & Casey, 2015), which could introduce significant heterogeneity and contribute to these inconsistencies in the literature.

The effects of pharmacological interventions on emotional processing and emotional regulation have been poorly characterised, particularly in young people. In adults, there is meta-analytic evidence suggesting that antidepressant treatment promotes the use of more adaptive strategies, leading to increased use of reappraisal and decreased use of suppression (McRae et al., 2014). Neurally, antidepressants have been shown to reduce the imbalance between limbic and prefrontal regions, by lowering activity in the amygdala and other areas responsible for the excessive processing of negative information, whilst enhancing the engagement of regions implicated in the cognitive regulation of emotions, including the DLPFC (for a review see Ma, 2015). In adolescents, there is evidence suggesting that antidepressants also reduce the processing of negative cues, particularly of anger (Capitão, Murphy, Browning, Cowen, & Harmer, 2015), however the effects of antidepressant drug treatment on emotional regulation specifically have not been investigated. In addition, studies in adolescents conducted to date are limited by the absence of a placebo control as well as the measurement of neural changes after relatively prolonged treatment durations. Concomitant changes in symptoms after long treatments make it difficult to determine whether fluoxetine has a direct effect on neural activity or whether these changes are an indirect consequence of mood improvement and/or treatment expectations.

This study therefore used fMRI to explore, for the first time, the effects of a single dose of fluoxetine on emotional processing as well as emotional reactivity and cognitive reappraisal in depressed adolescents aged 13 to 18 years. All patients recruited into the study had recently been prescribed fluoxetine by their psychiatrist for the treatment of depression. After being referred, adolescents with a confirmed diagnosis of MDD were then randomised to their first dose of fluoxetine 10 mg or placebo and were scanned using a battery of tasks, which included a well-validated emotional regulation paradigm (Phan et al., 2005; Reinecke et al., 2015). This task has been shown to reliably engage limbic and prefrontal circuitry in both adults and adolescents (Joormann & Stanton, 2016; Perlman et al., 2012), areas known to be sensitive to both the acute (Murphy, Norbury, O’Sullivan, Cowen, & Harmer, 2009; Rawlings, Norbury, Cowen, & Harmer, 2010) and long-term effects of antidepressants (Ma, 2015). We chose to administer the drug acutely in order to investigate the very early effects of fluoxetine against a placebo control and to avoid the confounding effects of concurrent changes in mood associated with long-term treatment. Part of the data from this study, focusing on emotional face processing, has been published elsewhere (Capitão et al., 2019). Given that emotion regulation abilities are still undergoing substantial development in adolescence (McRae et al., 2012), and may be particularly affected by depression (Ahmed et al., 2015), we also recruited a separate group of healthy adolescents who did not receive fluoxetine or placebo due to ethical reasons but completed the same emotional regulation task. We predicted that depressed participants on placebo (*v.* healthy controls) would show increased neural activation in both visual and limbic areas to aversive pictures in the Maintain *v.* Reappraise condition and that fluoxetine would reduce activity in these regions. Given that previous studies report inconsistent findings regarding the direction of PFC activity during cognitive reappraisal in adolescent depression (see Young et al., 2019), we did not formulate any *a-priori* hypothesis regarding activation in this region.

## Methods and materials

### Participants

Forty-eight participants (aged 13 to 18) were recruited. Of these, thirty-one were adolescent patients with a primary DSM-IV diagnosis of MDD and seventeen were healthy controls. Patients were recruited from Child and Adolescent Mental Health Services (CAMHS) from Oxfordshire, Reading and Milton Keynes and diagnoses were determined using the Schedule for Affective Disorders and Schizophrenia for School-Age Children-Present and Lifetime Version (K-SADS-P) (Kaufman et al., 1997). CAMHS psychiatrists made the clinical decision to initiate fluoxetine treatment and determined that it was safe for the patient to wait 2–7 days before initiating treatment in order to be able to participate in the study. Patients were excluded if they presented with (i) history of bipolar disorder or schizophrenia, (ii) substance abuse, (iii) current use of psychotropic/antidepressant medication, (iv) pregnancy or (v) MRI incompatibility (presence of metal implants or claustrophobia). A separate dataset using this sample of depressed adolescents has been previously published by our group (Capitão et al., 2019). Healthy adolescent controls with no history of psychological disorders were recruited from the community. Healthy controls were administered the same questionnaires and fMRI task, but did not receive any treatment due to ethical reasons.

This study was approved by the Southampton Research Ethics Committee (12/SC/0030). Participants aged 16 to 17 gave written informed consent. Participants younger than 16 provided written assent and their parent/guardian written consent.

A formal sample size calculation was precluded, because no prior study had determined the acute effect of fluoxetine on brain activity in depressed adolescents. Our previous work showed that acute fluoxetine reduced facial recognition of anger, with an effect size of 0.81 (Capitão et al., 2015). In a previous fMRI study, a single dose of the selective serotonin reuptake inhibitor (SSRI) citalopram was found to reduce amygdala activation with an effect size of 1.19 in healthy volunteers (Murphy et al., 2009). Informed by these data, an a priori sample size calculation for the current between-subjects design yielded *n* = 13 as the minimum sample size required to detect neural activity differences (difference between two independent means: two tailed, alpha = 0.05, effect size = 1.19, power = 0.8).

#### Procedures and measures

For a description of the measures used during screening, please refer to the online Supplementary Information.

Eligible patients were randomised to receive a single dose of either liquid fluoxetine (10 mg) or a matched placebo in a double-blind procedure. Placebo was peppermint syrup measured to the equivalent volume by a research psychiatrist not involved in the study (Chantiluke et al., 2014). Participants were asked to drink this mixture and then sat in a quiet room until the fMRI scan took place. The scan started 6 h after dosing, at a time where the plasma concentration of fluoxetine was expected to be at its peak (Rossi, Barraco, & Donda, 2004). Healthy controls did not receive any treatment, but completed the same scan as depressed adolescents.

Measures of state anxiety (STAI-CS) (Spielberger, 1973), mood (Visual Analogue Scale, adapted from Bond and Lader) (Bond & Lader, 1974) and side effects [Bodily Symptoms Checklist, adapted from Sinclair and colleagues (Sinclair et al., 2009)] were completed at three time points: before the drug/placebo administration, before the neuroimaging scan, and immediately after the scan. Healthy controls were administered the same measures but at only two timepoints: before and immediately after the scan.

After the testing session, patients were instructed to start fluoxetine treatment as prescribed by their treating psychiatrist, who also managed their subsequent care.

### Emotional regulation measures

#### fMRI task design

The emotion regulation task administered here was previously used by Reinecke et al. (Reinecke et al. 2015) and is an adaptation from the original paradigm developed by Phan and colleagues (Phan et al., 2005). This paradigm involved the presentation of aversive pictures from the International Affective Picture System (IAPS) (Lang, Bradley, & Cuthbert, 1997) across two main conditions of interest: Maintain (M) and Reappraisal (R). On Maintain (M) blocks, subjects were instructed to passively view the images and experience naturally (without trying to change) the emotional state elicited. On Reappraisal (R) blocks, subjects were instructed to decrease voluntarily the intensity of their negative affect by implementing cognitive strategies of reappraisal, i.e. using strategies such as reframing or rationalising to reinterpret the content of the picture so that it no longer elicits a negative response. Prior to the task, participants received instruction on cognitive reappraisal from a trained clinical psychologist (LC). The strategies of reframing and rationalising were exemplified: (a) reframing involves attributing a more positive connotation or meaning to a given scenario (e.g. woman crying in hospital could be interpreted as expressing joy after receiving good news rather than sadness) and (b) rationalising involves objectifying the content of the pictures (e.g. man with blood marks could be an actor wearing makeup for a movie rather than a victim of assault). These examples were provided for illustrative purposes, and participants were informed that they could select the most effective strategy for each picture. During training, participants were shown five images not used in the task and asked to practice using cognitive reappraisal while verbalising their reappraisal strategies. Feedback was provided when relevant. Participants were also instructed not to look away (unless necessary) or distract themselves with unrelated thoughts during the fMRI task. Subjects confirmed their understanding of the instructions and reappraisal strategy prior to scanning, as evidenced by their ability to provide detailed verbal descriptions of the techniques they would use to modulate their emotional experience.

Pictures were presented in eight blocks of five images. Picture blocks alternated with grey fixation baseline blocks, in which participants were asked to relax and clear their minds as much as possible. At the end of each block, participants indicated via keypad response the intensity of distress experienced throughout the block using a 4-point scale (1 = no distress to 4 = severe distress). After the task/scan, participants were shown eight pictures previously presented, and asked to recall and then write down the cognitive reappraisal strategies they had used for each picture.

#### Emotional regulation questionnaire (ERQ)

The ERQ (Gross & John, 2003) was administered at baseline to measure the adolescents' tendency to use the strategies of cognitive reappraisal and expressive suppression when regulating their emotions in their daily lives. As described earlier, cognitive reappraisal involves thinking differently about a situation in order to change one's emotional experience (e.g. *‘When I am feeling negative emotions* (*such as sadness or anger*), *I change what I am thinking about’*). Expressive suppression involves decreasing the external expression of an emotion (e.g. *‘When I am feeling negative emotions, I make sure not to express them’*). Cognitive reappraisal is associated with healthier patterns of social functioning and well-being, compared with expressive suppression (Cutuli, 2014).

#### Emotional regulation fMRI analysis

fMRI data were pre-processed and analysed using FEAT (FMRI Expert Analysis Tool), version 6.0.4, part of FSL (FMRIB's Software Library; www.fmrib.ox.ac.uk/fsl). For more information on the steps involved in data pre-processing and first-level and second-level analyses, please refer to the online Supplementary Information. Significant activations were identified using cluster-based thresholding of statistical images with a height threshold of Z > 3.1 and a (corrected) spatial extent threshold of *p* < 0.05 (Friston, Worsley, Frackowiak, Mazziotta, & Evans, 1993). At the whole-brain level, two regressors of interest [Maintain (M), Reappraise (R)] and two regressors of no interest (instruction/rating periods) were included. Fixation blocks were the implicit baseline reference (B). The analysis included the following comparisons: (a) Reappraise *v.* Maintain (R > M) to identify regions with greater activity during voluntary reduction of negative affect using reappraisal techniques; (b) Maintain *v.* Reappraise (M > R) to identify regions with more activity during passive viewing of aversive pictures; and (c) conjunction of effects across both Maintain and Reappraise (i.e. M + R > B), to identify neural regions that activate in both conditions.

These individual activation maps were then entered into the group level, which compared (a) depressed adolescent patients on placebo *v.* healthy controls, (b) depressed adolescent patients on fluoxetine *v.* placebo and (c) depressed adolescent patients on fluoxetine *v.* healthy controls, using a mixed-effects analysis across the whole brain. Significant interactions from whole-brain analyses were further explored by extracting BOLD parameter estimates.

#### Statistical treatment of demographic, clinical and subjective data

Questionnaire data were analysed by using IBM SPSS 22 software. Baseline characteristics (e.g. age, depression severity) were analysed by using analysis of variance (ANOVA), independent *t* tests or χ^2^ tests. The self-report scales used to assess the effects of fluoxetine were analysed using a mixed-design ANOVA, with timepoint (baseline, before scan and after scan) and group (fluoxetine *v.* placebo) as within- and between-subjects factors, respectively. The ERQ questionnaire administered at screening was also analysed using a mixed-design ANOVA (with two groups: depressed adolescents and healthy controls). The behavioural ratings from the fMRI task were also analysed using a mixed-design ANOVA, with 3 groups (depressed adolescents on placebo, depressed adolescents on fluoxetine and healthy controls). Significant interactions in the ANOVA were further explored using pairwise comparisons. When sphericity was violated, Greenhouse Geisser corrections were reported, but uncorrected degrees of freedom are reported for clarity. A *p* value lower than 0.05 was used to denote statistical significance. Marginal differences with a *p* value lower than 0.10 are also reported. Partial eta squared (*η*p^2^) and Cohen's d are reported as measures of effect size for ANOVAs and independent *t* tests, respectively.

## Results

### Demographic and baseline clinical characteristics

Demographic and baseline clinical characteristics are presented in Table 1. For additional information regarding the participant clinical characteristics please refer to Capitão et al. (Capitão et al. 2019). Of the 31 adolescent patients who were randomised, 2 were subsequently excluded due to the MRI not being successfully completed. Of the 17 healthy controls, 1 was excluded due to technical difficulties in recording behavioural data. The final sample therefore consisted of 45 participants (29 patients and 16 healthy controls).

**Table 1.** Demographic and clinical characteristics

	Placebo (*N* = 15)	Fluoxetine (*N* = 14)	Controls (*N* = 16)
Age	15.7 (1.4)	16.0 (1.2)	16.3 (1.1)
Female:male ratio	12:3	10:4	11:5
Ethnicity (Caucasian), *N* (%)	13 (86.7%)	14 (100%)	15 (93.8%)
IQ	114.7 (12.2)	111.6 (8)	119.6 (7)
Left:Right Handedness (ratio)	0:15	3:11	2:14
Family income level (median)	3 (1-6)	3 (1-6)	4 (1-6)
Family composition (intact), *N* (%)	8 (53.3%)	7 (50%)	10 (62.5%)
Depression severity			
CDI	32.8 (6)	29.8 (7.8)	4.8 (4.4)
CDRS-R	62.3 (8.6)	61.2 (13.3)	18.4 (2.4)
Trait anxiety	51.3 (4.3)	48.2 (5.2)	26.9 (4.2)
Suicidal thinking (SIQ-JR)	53.13 (25)	46.8 (21.4)	3.8 (3.6)
Emotional Regulation (ERQ)			
Cognitive reappraisal	21.5 (7.6)	20.5 (7)	27.5 (3.2)
Expressive suppression	19.2 (3.8)	18.6 (3.2)	12.56 (2.6)
Duration of MDD episode (months)	14.2 (8.7)	13.8 (11.1)	N/A
Mean age at onset of MDD (s.d.)	13.6 (2.2)	14.6 (1.4)	N/A
Mean number of MDD episodes (range)	1.3 (1-2)	1.1 (1-2)	N/A
Antidepressant naïve^a^, *N* (%)	12 (80%)	13 (92.9%)	N/A
Current psych. therapy/counselling, *N* (%)	9 (60%)	10 (71.4%)	N/A

*Notes:*

a All patients were antidepressant-naïve apart from 4 (3 in the placebo group and 1 in the fluoxetine group). 3 of these patients had received treatment with fluoxetine in the past (due to depression) and another patient in the placebo group was taking amitriptyline for the treatment of fibromyalgia immediately before starting fluoxetine. This patient stopped taking amitriptyline for a period of 4 days before the testing session. This washout period was considered appropriate given that amitriptyline has a mean elimination half-life of 20 h (ranging from 9 to 46 h). Family income per year was obtained using the following categories: 1 = Under £ 14 999; 2 = £ 15 000-£ 30 000; 3 = £ 30 000-£ 45 000; 4 = £ 45 000-£ 60 000; 5 = £ 60 000-£ 75 000; 6 = Above £ 75 000.

There were no significant differences between the 3 groups in age, gender distribution, ethnicity, family income or family composition (all *p*s>0.18). Healthy controls did however show higher IQ when compared to depressed adolescents (*p* = 0.037). As expected, healthy controls also showed reduced trait anxiety, depression scores and suicidal ideation at screening (*p* < 0.001).

The patient groups (fluoxetine *v.* placebo) were well matched in the baseline clinical measures such as mean age of depression onset, number of comorbidities, depression severity, trait anxiety and suicidal ideation (Table 1).

### Subjective ratings

As reported in our previous paper that assessed the effects of fluoxetine in the same patients using a different task (Capitão et al., 2019), no significant effect of fluoxetine *v.* placebo was seen on state anxiety or on any of the VAS scales (all *p*s>0.7). Side effects were measured using a non-validated scale given the lack of suitable measures available to investigate acute antidepressant drug effects. Participants receiving fluoxetine reported a significantly lower number of bodily symptoms across all time points, i.e. even at baseline (*F*(1,24) = 6.874, *p* = 0.015, 4.82 *v.* 2.97, *η*p2 = 0.223).

### Emotional regulation questionnaire (ERQ)

We compared the use of self-reported emotional regulation strategies between depressed adolescents and healthy controls, as assessed during the screening visit. There was a significant interaction between group and type of strategies used [*F*(1,42) = 34.975, *p* < 0.001, *η*p2 = 0.454]. Compared to healthy controls, depressed adolescents reported a lower use of cognitive reappraisal strategies [*F*(1,42) = 9.137, *p* = 0.004, *η*p2 = 0.179] and a higher use of expressive suppression [*F*(1,42) = 38.098, *p* < 0.001, *η*p2 = 0.476].

### Emotional regulation task

#### Behavioural ratings

During the task, participants were asked to indicate the intensity of distress experienced throughout the block using a keypad. These ratings were completed at the end of each block. Distress ratings were significantly lower in the Reappraise *v.* Maintain blocks [*F*(1,41) = 43.509, *p* < 0.001, *η*p2 = 0.515], without any between-group differences, therefore showing that both depressed and healthy adolescents were able to reduce negative affect during cognitive reappraisal.

Similarly, reaction times (RTs) were significantly faster in the Reappraise condition relative to Maintain [*F*(1,41) = 13.000, *p* = 0.001, *η*p2 = 0.476]. There was no significant interaction between condition and group (*p* > 0.79), although there was a trend for a main effect of group when considering RTs [*F*(1,41) = 2.720, *p* = 0.078, *η*p2 = 0.117], with healthy adolescent controls being slower overall than both patient groups.

Following the task, participants were shown eight pictures previously presented during Reappraise blocks and asked to recall the cognitive reappraisal strategies implemented for each. There were significant group differences [*F*(2,41) = 4.775, *p* = 0.014], with healthy adolescents reporting a higher number of cognitive reappraisal strategies than depressed adolescents on placebo (average of 7.31 ± 1.08 *v.* 5.0 ± 2.62, respectively; *p* = 0.005, cohen's d = 1.153), though this was only at trend level when compared to depressed adolescents on fluoxetine (average of 7.31 ± 1.08 *v.* 6.18 ± 2.36, respectively; *p* = 0.104). No significant differences were seen between depressed adolescents on placebo *v.* fluoxetine (average of 5 ± 2.62 *v.* 6.18 ± 2.36, respectively; *p* = 0.248).

#### fMRI analysis

##### Main effect of task

**Fig. 1.**
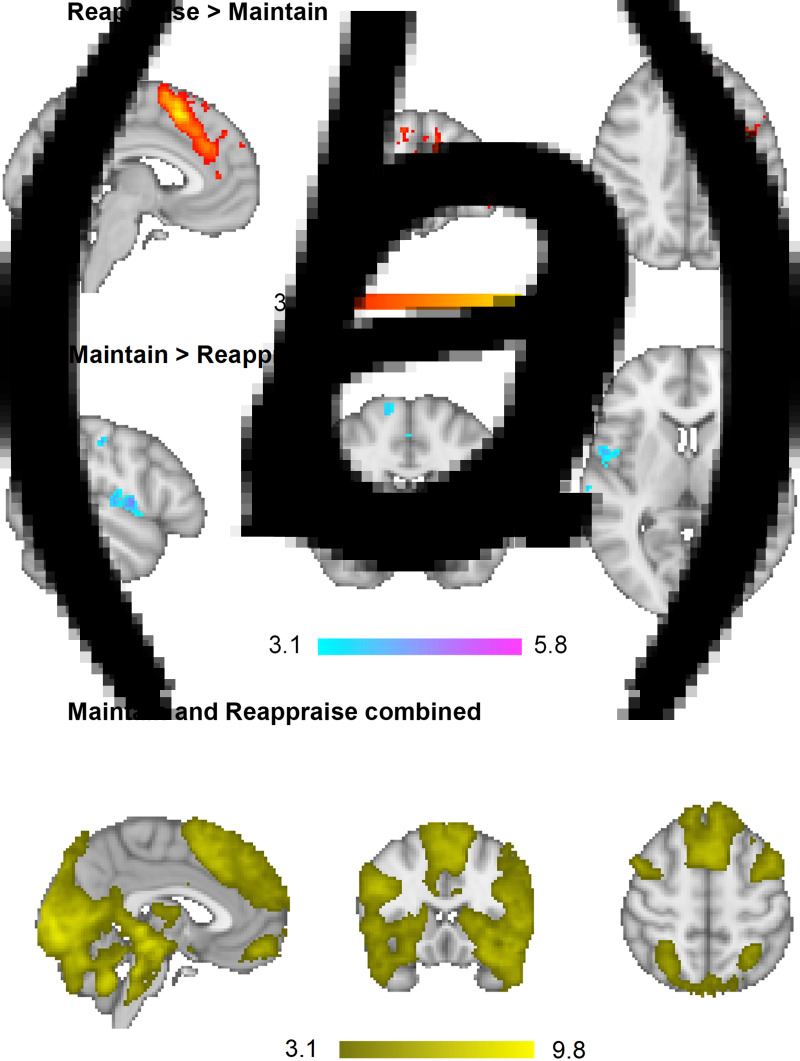
Main effect of task: across all three groups (*a*) Reappraise led to increased activation in clusters containing the superior, inferior and middle frontal gyrus, dorsal ACC, occipital fusiform cortex, cerebellum and middle/superior temporal gyrus relative to Maintain. (*b*) Maintain led to greater BOLD responses in the primary somatosensory cortex, including the precentral and postcentral gyrus, extending into the insula and inferior/medial frontal gyrus relative to Reappraise. (*c*) Across both conditions, there was significant activation in a large number of areas, with the peak clusters located in the occipital pole/fusiform gyrus as well as the frontal medial cortex, extending into the frontal pole. Whole-brain, images thresholded at Z > 3.1.

In order to identify which brain regions were *differentially* activated between conditions, Maintain and Reappraise conditions were compared across groups in a whole brain analysis. There was increased BOLD activation during Reappraise relative to Maintain in clusters containing the superior, inferior and middle frontal gyrus, dorsal ACC, occipital fusiform cortex, cerebellum and middle/superior temporal gyrus. In contrast, Maintain relative to Reappraise led to greater activity in the primary somatosensory cortex, including the precentral and postcentral gyrus, extending into the insula and inferior/medial frontal gyrus. Activation in these areas is consistent with previously published data in adults (Phan et al., 2005; Reinecke et al., 2015) and adolescents (McRae et al., 2012) (Table 2 and Fig. 1*a* and *b*).

**Table 2.** Whole-brain analysis results, images thresholded at Z > 3.1

Condition	Cluster	Side	Cluster size (voxels)	MNI (x,y,z)	Z-score	p-value
Main effect of task across groups						
Reappraise > Maintain	Superior frontal gyrus, extending into paracingulate and cingulate gyrus/dorsal ACC (BA 6, 32)	R/L	6916	−4, 16, 52	5.85	<0.001
	Cerebellum, extending into the occipital fusiform cortex	R	212	32, −64, −28	4.42	<0.001
	Inferior and middle frontal gyrus, extending into frontal pole (BA 45, 46)	R	189	56, 28, 14	4.66	<0.001
	Cerebellum	R	99	38, −56, −60	4.42	<0.05
Maintain > Reappraise	Precentral and postcentral gyrus/primary somatosensory cortex, central opercular cortex (BA 4, 6)	R	712	62, 2, 14	4.92	<0.001
	Central opercular cortex, extending into the inferior frontal gyrus and insula (BA 6, 22, 44, 13)	L	321	−50, 0, 6	4.47	<0.001
	Medial frontal gyrus, supplementary motor cortex, extending into the anterior ACC (BA 6)	R/L	183	−4, −8, 52	4.32	<0.001
	Superior frontal gyrus and precentral gyrus (BA 6)	R	126	14, −10, 70	4.41	<0.01
	Postcentral gyrus/primary somatosensory cortex (BA 2, 3)	L	126	−50, −20, 46	4.16	<0.01
Conjunction of Maintain and Reappraise *v*. baseline	Occipital pole, extending into lingual gyrus and occipital fusiform gyrus	L/R	102 147	14, −92, −4	9.87	<0.001
	Frontal medial cortex, extending into frontal pole	L/R	639	−2, 52, −16	6.33	<0.001

BA, Broadmann area; R, Right; L, Left.

Main effect of task across groups. MNI coordinates refer to the peak activation voxel of the cluster.

Lastly, in order to identify which brain regions were *activated across both conditions* (Maintain and Reappraise), brain activation in response to all negative pictures was compared to baseline. There was significant activation in a large number of areas, with the peak clusters located in the occipital pole/fusiform gyrus as well as the frontal medial cortex, extending into the frontal pole (Table 2 and Fig. 1*c*).

##### Effect of depression (depressed adolescents on placebo *v.* healthy controls)

**Fig. 2.**
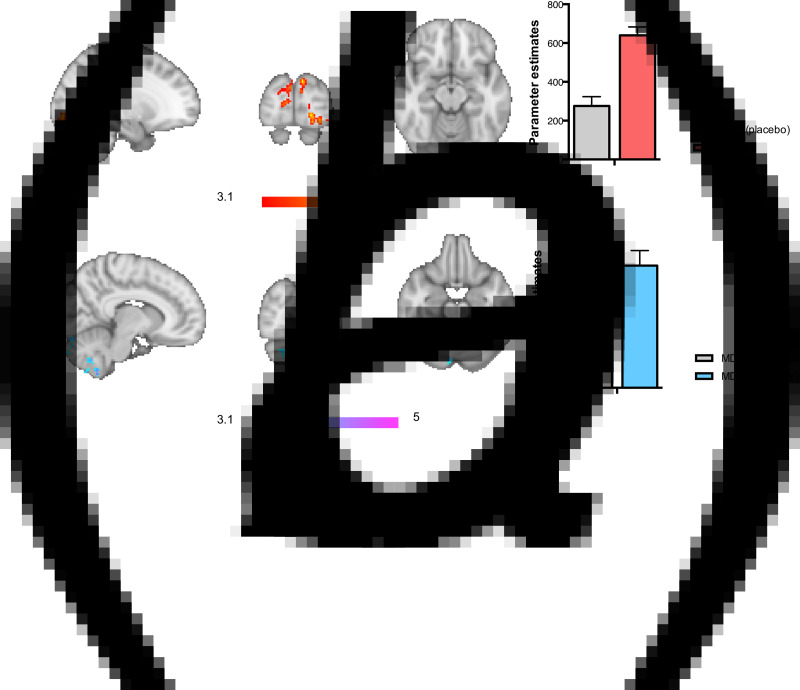
(*a*) Effect of depression: adolescent patients with MDD on placebo showed reduced activation in the occipital cortex and fusiform gyrus, compared to healthy controls across both Maintain and Reappraise conditions (*v*. baseline). (*b*) Effect of fluoxetine: in contrast, MDD patients on fluoxetine *v*. placebo showed increased activity in the cerebellum and occipital fusiform gyrus, also across both Maintain and Reappraise conditions (*v*. baseline). MDD refers to major depressive disorder, HC to Healthy Controls. Whole-brain, images thresholded at Z > 3.1.

There were no significant differences in BOLD activation between depressed adolescents on placebo and healthy controls in Reappraise compared with Maintain. When considering both Maintain and Reappraise conditions (*v.* baseline), depressed adolescents on placebo showed reduced activation in the occipital cortex and the fusiform gyrus to the negative picture stimuli when compared to healthy adolescents (Table 3 and Fig. 2*a*).

**Table 3.** Whole-brain analysis results, images thresholded at Z > 3.1. (a) Effect of depression; (b) Effect of treatment

(*a*) *Effect of depression* (*healthy adolescent controls higher than depressed adolescentson placebo*)						
Maintain > Reappraise	–	–	–	–	–	–
Reappraise > Maintain	–	–	–	–	–	–
Conjunction of Maintain and Reappraise *v*. baseline						
	Occipital cortex/occipital pole (BA 18), extending into the fusiform gyrus	L	330	−18, −90, −6	4.39	<0.001
	Occipital pole/intracalcarine cortex (BA 18)	R	177	4, −98, 14	3.84	<0.01
(*b*) *Effect of treatment* (depressed adolescents on *fluoxetine higher than depressed adolescents on placebo*)						
Maintain > Reappraise	–	–	–	–	–	–
Reappraise > Maintain	–	–	–	–	–	–
Conjunction of Maintain and Reappraise *v*. baseline	Cerebellum extending into lingual gyrus/occipital fusiform gyrus	R	150	8, −54, −60	4.35	<0.01
	Cerebellum	R	138	38, −68, −40	4.16	<0.01
	Cerebellum	R	128	14, −86, −36	3.99	<0.05
	Cerebellum	R	101	52, −62, −50	4.62	<0.05
	Cerebellum	L	95	−46, −60, −54	4.3	<0.05

BA, Broadmann area; R, Right, L, Left.

MNI coordinates refer to the peak activation voxel of the cluster.

##### Effect of treatment (depressed adolescents on placebo *v.* fluoxetine and depressed adolescents on fluoxetine *v.* healthy controls)

There were no significant differences between the placebo and the fluoxetine groups in BOLD activation in Reappraise compared with Maintain. When considering both Maintain and Reappraise conditions (*v.* baseline), depressed adolescents on fluoxetine showed significantly increased BOLD activation compared with the placebo group in clusters containing the cerebellum and lingual gyrus/occipital fusiform gyrus to the negative picture stimuli (Table 3 and Fig. 2*b*). No significant differences were seen in the contrast comparing depressed adolescents on fluoxetine *v.* healthy controls.

## Discussion

In this study, we implemented a previously validated emotional regulation paradigm in order to investigate the neural effects of a single dose of fluoxetine *v.* placebo on emotional reactivity and cognitive reappraisal in a sample of depressed adolescents. Contrary to our hypothesis, we did not see any significant differences in limbic or PFC activity in adolescents with depression *v.* healthy controls when comparing Maintain *v.* Reappraise. Rather, depressed adolescents showed reduced visual activation to aversive pictures irrespective of the condition when compared to healthy controls. Fluoxetine was shown to reverse this pattern, as depressed adolescents on fluoxetine (*v.* placebo) showed increased activity in visual processing areas and in the cerebellum in response to both conditions.

### Effects of depression

This sample of depressed adolescents was not significantly different from healthy controls in their ability to modulate frontal neural activation and implement the strategy of cognitive reappraisal. The main effect of the task revealed that the Reappraise condition (when compared to Maintain) led to reduced distress ratings, as well as increased frontal activity, including in the superior frontal cortex and dorsal ACC (dACC), areas implicated in the cognitive control of emotional stimuli (Bush, Luu, & Posner, 2000; Giuliani, Drabant, & Gross, 2011), without any significant differences between the groups. These findings are consistent with data showing that adolescents with depression are equally able as healthy controls to increase frontal cortical activity during instructed cognitive reappraisal (Del Piero, Saxbe, & Margolin, 2016; Herres, Ewing, & Kobak, 2016, but see Young et al., 2019) and to reduce their levels of distress following the implementation of relevant cognitive restructuring techniques (Platt et al., 2015). However, an analysis of self-report data from the Emotional Regulation Questionnaire administered during the screening visit showed that these groups differed in their daily life use of emotional strategies. When compared to healthy controls, depressed adolescents reported a less frequent use of cognitive reappraisal strategies and a more frequent use of expressive suppression. It is possible that adolescents with depression have the ability to implement strategies of cognitive reappraisal after receiving specific instructions and relevant training, but struggle to implement them spontaneously in their daily lives.

A combination analysis of the Maintain and Reappraise conditions showed reduced activity in the occipital cortex and fusiform gyrus in the depressed adolescent group on placebo, when compared to healthy controls. This was in contrast to our hypothesis, as we predicted that depressed adolescents would show increased visual and limbic activity in response to aversive pictures (Perlman et al., 2012). The occipital cortex and fusiform gyrus are involved in early visual processing and attentional processes (Cohen, 2014; Somers & Sheremata, 2013), and are part of a detection node of social stimuli particularly relevant in adolescence (Nelson, Leibenluft, McClure, & Pine, 2005; Stephanou et al., 2016). It is therefore possible that despite being instructed not to look away from the pictures unless necessary, the depressed group avoided the pictures more (by diverting or closing the eyes or by looking at non-emotional features of the stimuli), resulting in lower activation in the occipital cortex and occipital fusiform gyrus. Despite changes in visual cortex activity not being typically associated with depression, there is growing evidence implicating this region in the disorder (Beauregard et al., 1998; Furey et al., 2013; Keedwell et al., 2010) which may track differences in attention i.e. hypervigilance and avoidance of aversive cues. Consistent with this, there is research suggesting that depression in adolescence is frequently characterised by experiential avoidance (Mellick et al., 2019), i.e. an unwillingness to remain in contact with painful thoughts, experiences or reactions. Whilst avoidance can allow the individual to redirect attention away from images, thoughts or feelings that are negative or too painful - hence providing immediate escape from the situation - it is not an optimal strategy in the long-term, as it takes away the possibility to learn alternative strategies to deal with the feared stimuli, hence reinforcing the depressive state. Indeed, higher levels of threat avoidance have been shown to predict greater 2-year depression scores in young people (Price et al., 2016).

Interestingly, and as mentioned previously, self-report data collected at baseline showed that adolescents with depression reported a higher use of expressive suppression, which refers to a tendency to hide, inhibit or reduce ongoing emotion-expressive behaviour (Gross & John, 2003; Gross & Levenson, 1993). Expressive suppression is generally considered an ineffective strategy of emotional regulation and has been associated with depression in both adult (Gross & John, 2003; John & Gross, 2004) and adolescent samples (Betts, Gullone, & Allen, 2009; Larsen et al., 2013). Whilst the current task was not designed to measure emotional suppression or avoidance *per se*, it is possible that these processes could have influenced neural responsivity to the negative pictures included. It is also important to note that adolescence is a period during which other affective processes, such as mentalising – i.e. the ability to make sense and reflect about the actions of ourselves and others on the basis of intentional mental states, such as desires, feelings, and beliefs (Fonagy & Allison, 2012) - continue to develop, which may be of relevance to the adolescents' processing of the stimuli used in this study, many of which had a social context (e.g. depicting people in distress). Future research in emotional regulation is needed, combining eye tracking – a method that would provide a direct and objective measure of attention allocation – as well as measures of social cognition such as theory of mind and mentalising.

### Effects of fluoxetine

Depressed adolescents on fluoxetine (*v.* placebo) showed a pattern of increased neural activity in the cerebellum and occipital cortex/fusiform gyrus also in response to both Maintain and Reappraise conditions, which was opposite to that seen in depressed adolescents on placebo *v.* healthy controls. Previous studies have also reported early effects of SSRIs on both the occipital cortex and fusiform gyrus (Grady et al., 2013; Norbury, Mackay, Cowen, Goodwin, & Harmer, 2007; Rawlings et al., 2010), in line with their role in attentional processing. Whilst the cerebellum has been traditionally involved in motor control, there is evidence suggesting that this neural region supports various cognitive functions, including attention (Allen & Courchesne, 1998; Gottwald, Mihajlovic, Wilde, & Mehdorn, 2003; Kellermann et al., 2012), and there is evidence implicating this region in depression (Beauregard et al., 1998; Li et al., 2013). Based on these data, it is possible that fluoxetine acts to increase attention to aversive stimuli, possibly via a reduction in avoidance. These findings are consistent with data by Di Simplicio and colleagues (Di Simplicio et al., 2014a; 2014b), who showed that treatment with the SSRI citalopram increased neural activity to threatening facial images in a sample of highly neurotic adult participants. Intriguingly, these authors also found that citalopram reversed a pattern of ocular avoidance of facial stimuli (Di Simplicio et al., 2014a, 2014b), hence lending support to the hypothesis that SSRI treatment may act to reduce emotional avoidance in vulnerable populations. This may be particularly helpful for adolescents with depression, who often avoid unpleasant cues and social situations (Fernández-Theoduloz et al., 2019; Mellick et al., 2019; Young et al., 2019).

The findings from this study are important, as they provide the first evidence that fluoxetine modifies neural circuitry relevant to emotional regulation in young people. The effects of antidepressants have been significantly under-studied in young people and there is an urgent need to conduct research in this area (Murphy et al., 2021). A separate dataset from this study (published elsewhere, Capitão et al., 2019), demonstrated that a single dose of fluoxetine reduces limbic responses to anger, and increases activity in the dorsal anterior cingulate cortex. The data from the current paradigm adds to this evidence, by suggesting that fluoxetine may act to reduce avoidance to aversive images. Similar to treatments such as cognitive behavioural therapy (CBT), fluoxetine could help the adolescent redirect attention to painful stimuli and thoughts hence creating an opportunity for them to learn alternative strategies to handle situations previously avoided. Due to the absence of eye-tracking and the explorative nature of this study, these data and interpretations should nonetheless be treated as preliminary.

In addition, whilst these effects of fluoxetine are being interpreted as reduced visual avoidance of aversive images, we cannot exclude the possibility that increased neural activity towards negative stimuli reflects a dysfunctional effect of SSRI administration, which could help explain the pattern of increased anxiety often experienced early in treatment with SSRIs (Browning, Reid, Cowen, Goodwin, & Harmer, 2007; Licinio & Wong, 2005). This interpretation has also been considered in previous studies (e.g. Di Simplicio et al., 2014a, 2014b). The fact this pattern induced by fluoxetine was similar to that seen in healthy adolescent controls (*v.* depressed patients) argues against this hypothesis, as does our previous data showing that a single dose of fluoxetine has anxiolytic-like effects in young adults aged between 18 and 21 (Capitão et al., 2015). Nevertheless, it is possible that the nature of the aversive images used here could have elicited, to some extent, an anxiogenic reaction in this population of adolescent patients following fluoxetine administration. This possibility needs to be clarified in future studies, especially combining eye-tracking methodologies and fMRI, and using task paradigms specifically designed to investigate different attentional processes, including avoidance and disengagement.

### Limitations

Several limitations of this study should be noted. Firstly, although we did a power calculation, our sample size was relatively small. Future studies with a larger cohort of patients are important to replicate and further expand these findings. Secondly, a range of ages was included in this study, and it is known that emotional regulation changes substantially during adolescence. It would be important for future work to address adolescent stage as a moderator of emotional regulation and antidepressant drug effects. Thirdly, this study only included negative/aversive pictures, but future studies should also include positive and neutral stimuli to determine whether the effects seen here are specific to negative images. Fourthly, we did not collect eye-tracking which complicates interpretations about how gaze and attentional processes could have contributed to the neural patterns reported here. Future studies would benefit from the addition of eye-tracking and arousal measures (such as heart rate and skin conductance). Finally, the task included general aversive images that were not specific to adolescence. Therefore, it would be important for future studies to select real-world scenarios that are relevant to this age group (such as those including scenes of bullying/ostracism; peer conflict, etc.), as these may evoke stronger behavioural and neural responses.

## Conclusion

To conclude, this is the first time that the effects of fluoxetine on the neural substrates underlying emotional regulation are being investigated in a sample of depressed adolescents. Contrary to expectations, depressed adolescents on placebo (*v.* healthy controls) showed reduced (rather than increased) visual activity in response to aversive pictures, and fluoxetine was found to increase visual activity to both Maintain and Reappraise conditions. These data tentatively suggest that fluoxetine may act to reduce avoidance to aversive pictures, hence highlighting a potentially important, and complementary, mechanism of fluoxetine. This hypothesis nonetheless needs to be further explored in future studies using eye-tracking in addition to fMRI.

## Supplementary material

For supplementary material accompanying this paper visit https://doi.org/10.1017/S0033291722001805. click here to view supplementary material
