# Supplementary material for: Acute neural effects of fluoxetine on emotional regulation in depressed adolescents
Source: Psychol Med. 2022 Jul 29;53(10):4799–810. doi: 10.1017/S0033291722001805 (PMC10388313; doi:10.1017/S0033291722001805)
Supplement: Supplementary file 1 [file S0033291722001805sup001.docx]

**Supplementary Methods**

Procedures and measures

Psychiatric diagnoses were determined using the Schedule for Affective Disorders and Schizophrenia for School-Age Children-Present and Lifetime Version (K-SADS-P), administered either by a Child and Adolescent Psychiatrist (RB) or a Clinical Psychologist (LC). Depression severity was assessed using the Child Depression Inventory (CDI) and the Childhood Depression Rating Scale-Revised (CDRS-R). Participants were also administered the State-Trait Anxiety Inventory for Children (STAI-C) and the Suicidal Ideation Questionnaire-Junior version (SIQ-JR). Parents/guardians completed the Child Behaviour Checklist (CBCL). IQ scores were determined using the 2-subscale version of the Wechsler Abbreviated Scale of Intelligence (WASI). Healthy controls were administered the same measures.

fMRI data analysis

Functional imaging consisted of 45 T2-weighted echoplanar imaging (EPI) slices (TR=3000ms, TE=30ms, matrix 64 x 64, slice thickness=3mm), 3mm^3^ voxels. An anatomical image (TR=2040ms, TE=4.7ms), 1mm^3^ voxels, was also acquired to allow later registration of the fMRI data into standard space.

Pre-processing involved a number of steps designed to reduce unwanted variability in the data and to improve the validity of statistical analyses: motion correction using FMRIB’s Linear Image Registration Tool (MCFLIRT; Jenkinson, Bannister, Brady, & Smith, 2002); deletion of non-brain tissue using the Brain Extraction Tool (BET; Smith, 2002); spatial smoothing with a Gaussian kernel of 5 mm full-width-half-maximum; grand-mean intensity normalisation of the entire 4D dataset by a single multiplicative factor; high pass temporal filtering (Gaussian-weighted least-squares straight line fitting, with sigma of 45s) and B0 unwarping using fieldmap phase and magnitude images for distortion correction. In addition, registration to high-resolution image and to a standard template [Montreal Neurological Institute (MNI)] was implemented using FNIRT nonlinear registration (Andersson, 2007).

In the first-level analysis, individual activation maps were computed using the general linear model with local autocorrelation correction. Three explanatory variables were modelled: “Maintain”, “Reappraise” and “Instructions/Ratings”. Temporal derivatives were included in the model as covariates of no interest to increase statistical sensitivity. Variables were modelled by convolving each block with a haemodynamic response function, using a variant of a gamma function (i.e. a normalisation of the probability density function of the gamma function) with a standard deviation of 3s and a mean lag of 6s.

In the second-level analysis, whole-brain individual data were combined at a group level using a mixed-effects group cluster analysis across the whole brain corrected for multiple comparisons. Such a mixed-effects approach accounts for intra-subject variability and allows general population inferences to be drawn.

At the whole-brain level, Maintain and Reappraise blocks were compared with each other, resulting in the following model: 1) Maintain vs. fixation; 2) Reappraise vs. fixation; 3) Maintain>Reappraise; 4) Reappraise>Maintain; 5) combination of Reappraise and Maintain. Groups were also contrasted with each other, resulting in the following comparisons of interest: 1) placebo>fluoxetine; 2) fluoxetine>placebo; 3) healthy controls>depressed patients on placebo; 4) depressed patients on placebo>healthy controls. Significant interactions from whole-brain analyses were further explored by extracting BOLD parameter estimates for each emotion.

**Supplementary References**

Andersson, M. Jenkinson and S. Smith (2007) Non-linear registration, aka Spatial normalisation. FMRIB technical report TR07JA2 from www.fmrib.ox.ac.uk/analysis/techrep.

Capitão LP, Chapman R, Murphy SE, Harvey C-J, James A, Cowen PJ, et al. A single dose of fluoxetine reduces neural limbic responses to anger in depressed adolescents. Transl Psychiatry. 2019 Jan 21;9(1):30.

Jenkinson M, Bannister P, Brady M, Smith S (2002). Improved optimization for the robust and accurate linear registration and motion correction of brain images. *NeuroImage* 17, 825–841.

Smith SM (2002). Fast robust automated brain extraction. *Human Brain Mapping* 17, 143–155.

Woolrich MW, Behrens TEJ, Beckmann CF, Jenkinson M, Smith SM (2004). Multilevel linear modelling for FMRI group analysis using Bayesian inference. *NeuroImage* 21, 1732–1747.

Woolrich MW, Ripley BD, Brady M, Smith SM (2001). Temporal autocorrelation in univariate linear modeling of FMRI data. *NeuroImage* 14, 1370–1386.
